# Protect the oceans from Japan's radioisotope dumping

**DOI:** 10.1016/j.ese.2023.100369

**Published:** 2023-12-18

**Authors:** Chengjun Li, Huan Zhong, Lingyu Meng, Mengjie Wu, Wenjing Ning, Su Shiung Lam, Jun Luo, Christian Sonne

**Affiliations:** Institute of Environmental Research at Greater Bay Area, Guangzhou University, Guangzhou 510006, China; School of Environment, Nanjing University, Nanjing, China; Department of Civil Engineering, Nagoya Institute of Technology, Japan; Key Laboratory of Soil Environment and Pollution Remediation, Institute of Soil Science, Chinese Academy of Sciences, Nanjing, China; School of Environment, Nanjing University, Nanjing, China; School of Environment, Nanjing University, Nanjing, China; Universiti Malaysia Terengganu, Terengganu, Malaysia; Center for Global Health Research (CGHR), Saveetha Institute of Medical and Technical Sciences (SIMATS), Saveetha University, Chennai, India; School of Environment, Nanjing University, Nanjing, China; Aarhus University, Roskilde, Denmark; Sustainability Cluster, School of Engineering, University of Petroleum & Energy Studies, Dehradun, Uttarakhand, India

## Abstract

• Dumping of Fukushima's radioactive wastewater raises marine food web concern.
• Tritium seems to be the most problematic compound.
• Long-lived radioisotopes Biomagnify up to 50,000 folds in marine fish species.
• This threatens fragile deep-sea ecosystems requiring immediate action.
• Empowered Routine monitoring is crucial to maintain planetary health.

In August 2023, Japan started to dump its 1.3 million tons of radioactive wastewater into the oceans [1,2]. Most radioisotopes are claimed to be removed from the wastewater except tritium, which needs further dilution before discharge. However, this dilution does not effectively remove any tritium from the wastewater but serves as a measure to bring its abnormally high concentration down to meet emission standards. The most worrisome situation is that many more bioaccumulative long-lived radioisotopes (BLLRs), such as carbon-14 and cobalt-60, slip through the treatment process. There are attempts to reduce the concentrations of BLLRs via repurification to meet the regulatory standards [3]; however, even low levels of these BLLRs can undergo biomagnification of up to 50,000 folds in marine fish species [4].

Such long-term overexposure imposes risks to predatory fish and marine mammals, many of which are on the IUCN Red List as endangered or critically endangered [5]. In the Pacific deep sea for example being a sanctuary for marine species, BLLRs become increasingly bioavailable [6] and super-enriched by up to 50,000,000 folds in marine particles and sediments, threatening deep-sea biota and habitats [7]. Moreover, climate change further exacerbates BLLRs’ bioaccumulation and amplifies adverse impacts on marine biodiversity by altering seawater chemistry and increasing BLLRs' bioavailability and exposure to wildlife. This is driven by shifts in ocean currents and the transportation of enriched particles, both vertically and horizontally, as global warming could make seawater less cold and less likely to sink [[8], [9], [10]].

To address this critical issue, urgent actions are needed to establish global frameworks for systematic sampling and continuous monitoring of discharged BLLRs, covering seawater and organisms at various trophic levels. To empower the proposed initiatives, new equipment and techniques should be developed. These include, for example, long-endurance unmanned marine vehicles equipped with diffusive gradients in thin films, enabling large-scale routine monitoring of multiple BLLRs. The simultaneous improvement of existing modeling methods and development of new ones should likewise be another focal point of future research. This ensures a better understanding and prediction of BLLRs' dynamics in the ocean, both in seawater and along food chains. Such efforts have the potential to advance the understanding of BLLRs’ bioaccumulation, thereby mitigating BLLRs' risks and safeguarding invaluable marine ecosystems.

More importantly, to proactively and effectively minimize the impact of Japan's dumping of nuclear wastewater, comprehensive management measures must be implemented [11]. These measures should include, but not be limited to, implementing containment measures to prevent further releases, mitigating sources of contamination, exploring and deploying advanced water purification technologies. In addition, managing filtration systems and sediments, all help to address the persistent challenge posed by the released radioactive substances in the ocean.

## Declaration of competing interest

The authors declare that they have no known competing financial interests or personal relationships that could have appeared to influence the work reported in this paper.
